# Effect of partial supplementation of sun-dried *Azolla* as a protein source on the immunity and antioxidant status of commercial broilers

**DOI:** 10.14202/vetworld.2015.1126-1130

**Published:** 2015-09-23

**Authors:** Biswal Chichilichi, G. P. Mohanty, S. K. Mishra, C. R. Pradhan, N. C. Behura, A. Das, K. Behera

**Affiliations:** 1 Department of Livestock Production and Management, College of Veterinary Science & Animal Husbandry, Orissa University of Agriculture and Technology, Bhubaneswar, Odisha, India; 2 Department of Animal Nutrition, College of Veterinary Science & Animal Husbandry, Orissa University of Agriculture and Technology, Bhubaneswar, Odisha, India; 3 Postgraduate Department of Poultry Science, College of Veterinary Science & Animal Husbandry, Orissa University of Agriculture and Technology, Bhubaneswar, Odisha, India

**Keywords:** antioxidant, *Azolla*, broiler, immune response

## Abstract

**Aim:**

The present study was conducted to evaluate the effect of partial supplementation of sun-dried *Azolla* as a protein source on the immunity of commercial broilers in coastal Odisha.

**Materials and Methods:**

A 180 day-old broiler chicks were distributed in six dietary treatments *viz*. C_1_: Basal diet, C_2_: Basal diet + enzyme, T_1_: Basal diet +5% protein from *Azolla*, T_2_: Basal diet + 5% protein from *Azolla* + enzyme, T_3_: Basal diet +10% protein from *Azolla*, and T_4_: Basal diet + 10% protein from *Azolla* + enzyme. Cutaneous basophilc hypersensitivity (CBH) and humoral immunity response were determined at the 38^th^ day of age. At 42^nd^ day, the weight of lymphoid organs, an antioxidant enzyme, and lipid peroxidation activity were determined.

**Results:**

The CBH response did not differ significantly among the treated groups, but the sheep red blood cells response was significantly higher in T_4_. The weight of lymphoid organs or immune organs of all the treated groups did not differ significantly (p>0.05). The erythrocyte catalase level of T_4_ group was found to be significantly higher than rest of the treated groups except T_3_.

**Conclusion:**

It may be concluded that supplementation of *Azolla* at 10% of dietary protein requirement along with enzyme supplementation in an isonitrogenous diet showed a better immune response in broilers.

## Introduction

*Azolla* is, a protein rich aquatic plant, containing almost all essential amino acids, carotene, and several growth promoter intermediaries, minerals such as calcium, phosphorus magnesium, potassium, iron, and copper [1], and certain compounds such as carotenoids, bio-polymers, and probiotics [2]. In case of commercial broiler chickens, *Azolla* can be efficiently used as a feed ingredient in the form of sun-dried and ground *Azolla* meal [3].

*Azolla* meal can partially replace the dietary protein sources up to 5-10% without any adverse effect on the health and performance of the birds [4]. Inclusion of *Azolla* in the poultry diet helps in the economization of production cost; and thus, increasing the net profit [1]. Similar findings have been observed in the case of quails with optimum displacement level of *Azolla* restricted to 5% [5]. The high carotene content of *Azolla* is responsible for its immune-potentiating effect in poultry birds [1]. In non-ruminants, essential amino acids, linoleic acid, vitamin A, folic acid, vitamin B_6_, vitamin B_12_, vitamin C, vitamin E, zinc, copper, iron, and selenium affect one or more indices of immunity [6]. Use of *Azolla* as an antibacterial and antioxidant agent in complementary and alternate medicine [7] had been recommended due to its high phenolic and flavonoid content [8]. Antioxidant activity of *Azolla* had been successfully demonstrated in Swiss albino mice [9].

With consideration of the presence of essential nutrients in *Azolla*, the present experiment was planned with the objective of studying the effect of partial supplementation of sun-dried *Azolla* as a protein source on the immunity of commercial broilers.

## Materials and Methods

### Ethical approval

The experiment followed the guidelines of Institutional Animal Ethics Committee.

### Study area

The experiment was conducted on commercial broilers reared in the Poultry Farm in Department of Poultry Science, College of Veterinary Sciences and Animal Husbandry, Orissa University of Agriculture and Technology, Bhubaneswar, Odisha.

### Experimental birds

The 180 day-old broiler chicks were distributed randomly in six dietary treatments with three replications. The six dietary treatments were: C_1_: Basal diet, C_2_: Basal diet + enzyme, T_1_: Basal diet + 5% dietary protein from *Azolla*, T_2_: Basal diet + 5% dietary protein from *Azolla* + enzyme, T_3_: Basal diet + 10% dietary protein from *Azolla*, and T_4_: Basal diet + 10% dietary protein from *Azolla* + enzyme. The enzyme used in the study was containing cellulase, xylanase, pectinase, and phytase. The experiment continued up to 6^th^ week of age. The chemical composition of *Azolla* meal, ingredient composition and chemical composition of the experimental diet are provided in Tables-1, 2 and 3, respectively.

**Table-1:** Chemical composition of *Azolla* meal.

Nutrients	Percentage
Dry matter	91.03
Crude protein	25.42
Crude fiber	14.22
Ether extract	2.58
Total ash	18.75
NFE	39.02
Calcium	1.12
Phosphorus	0.53
Zinc (ppm)	159.1
Copper (ppm)	7.35
Manganese (ppm)	84.2
Iron (ppm)	284.7

NFE=Nitrogen free extract

**Table-2:** Ingredient composition of the experimental diet.

Ingredients	C_1_	C_2_	T_1_	T_2_	T_3_	T_4_
Starter ration						
Maize	55.0	55.0	54.0	54.0	50.3	50.3
Soybean meal	39.5	39.5	37.9	37.9	36.0	36.0
De-oiled rice bran	2.5	2.5	0.0	0.0	0.0	0.0
Oil	0.0	0.0	0.5	0.5	1.5	1.5
*Azolla*	0.0	0.0	4.6	4.6	9.2	9.2
Mineral mixture and common salt	3.0	3.0	3.0	3.0	3.0	3.0
Total	100	100	100	100	100	100
Enzyme	-	+	-	+	-	+
Finisher ration						
Maize	60.0	60.0	60.0	60.0	57.25	57.25
Soybean meal	31.0	31.0	29.5	29.5	28.0	28.0
De-oiled rice bran	5.25	5.25	2.5	2.5	2.0	2.0
Oil	0.75	0.75	1.0	1.0	1.75	1.75
*Azolla*	0.0	0.0	4.0	4.0	8.0	8.0
Mineral mixture and common salt	3.0	3.0	3.0	3.0	3.0	3.0
Total	100	100	100	100	100	100
Enzyme	-	+	-	+	-	+

**Table-3:** Chemical composition (percentage of dry matter basis) of experimental diet.

Nutrient	Starter ration			Finisher ration		
	
	C_1_/C_2_	T_1_/T_2_	T_3_/T_4_	C_1_/C_2_	T_1_/T_2_	T_3_/T_4_
Moisture	11.0	12.2	12.8	13.14	12.90	13.25
Crude protein	23.17	23.28	23.15	20.14	20.08	20.12
Ether extract	1.05	1.38	1.53	1.57	1.80	2.01
Crude fiber	4.01	4.58	4.92	4.03	4.30	4.86
Total ash	6.34	7.44	7.87	6.37	7.21	7.86
NFE	65.43	63.32	62.53	67.89	66.61	65.15
Calcium	0.92	1.08	1.14	0.82	0.91	0.91
Available phosphorus	0.48	0.51	0.50	0.53	0.51	0.54
Metabolizable energy*(Kcal/kg)	2800.50	2803.42	2809.26	2900.00	2899.78	2902.21

NFE=Nitrogen free extract

### Evaluation of immunological parameters

At 38^th^ day of age, two birds from each replicate in each dietary treatment were injected intradermally in the comb with 100 µg of phytohemaglutinin-P in 0.1 ml of normal saline to measure the cell-mediated immune (CMI) response by cutaneous basophilc hypersensitivity (CBH) test [10]. The thickness of comb was measured using digital caliper before inoculation and 24 h post inoculation, and CBH response was calculated as per Soni *et al*. [11]. At 38^th^ day, the measure of humoral immunity was carried out as per the method described by Abdallah *et al*. [12]. At 6^th^ week, three birds from each treatment were slaughtered and the weights of bursa, thymus, and spleen were recorded. About 3 ml blood, for assessing the antioxidant indices, was collected in sterilized microcentrifuge tubes containing acid citrate dextrose (citric acid 8.0 g, sodium citrate 22.0 g, and dextrose 25.0 g and volume made to 1 L in distilled water) at 0.15 ml/ml blood as anticoagulant. The blood samples were centrifuged at 3000 rpm for 10 min at 4°C and then the plasma and buffy coat were separated. The resulting erythrocyte pellet was washed thrice with phosphate buffer saline (PBS; pH 7.4; disodium hydrogen phosphate 13.65 g, sodium dihydrogen phosphate 2.43 g, and sodium chloride 10 g dissolved in 800 ml distilled water, pH adjusted to 7.4 and volume made to 1 L) as per Yagi *et al*. [13]. Red blood cell (RBC) diluted to 1:1 in PBS was used for the estimation of hemoglobin. For the estimation of catalase 1 ml of the 1:1 diluted RBCs in PBS were mixed with 9 ml distilled water to prepare hemolysate of 1:20 dilution. Erythrocyte catalase was assayed in erythrocytes by the spectrophotometric method as described by Bergmeyer [14]. The lipid peroxide level in the RBC hemolysate was determined by the method of Placer *et al*. [15].

### Statistical analysis

The data from the experiment were subjected to statistical analysis as per the methods suggested by Snedecor and Cochran [16].

## Results

### CBH and humoral immunity response

The CBH response and antibody titers (log_2_) against sheep RBCs (SRBC) inoculation at 6^th^ week of age of broiler chicks is presented in Table-4. The CBH response did not differ significantly among the treated groups. However, the SRBC response was significantly higher in T_4_ than that of other treated groups. The SRBC response of the birds of T_2_ was significantly higher than that of C_1_ but did not differ significantly (p>0.05) from other treated groups except T_4_.

**Table-4:** SRBC and CBH response of broiler chicks at 6 weeks of age.

Parameters (unit)	Treatments						p value

	C_1_	C_2_	T_1_	T_2_	T_3_	T_4_	
SRBC (log_2_)	6.67^c^±0.33	7.00^bc^±0.58	7.33^bc^±0.33	8.00^b^±0.58	7.67^bc^±0.33	9.67^a^±0.33	0.005
CBH	124.49±2.52	111.67±2.67	120.06±3.25	116.15±2.17	107.70±4.48	121.84±8.32	0.14

Values bearing different superscripts in a row differ significantly (p<0.05), SRBC=Sheep red blood cells, CBH=Cutaneous basophilc hypersensitivity

### Lymphoid organs

The weights of lymphoid organs (percentage of live weight) of Vencobb broiler chicks under different dietary treatments are presented in Table-5. The average weights of lymphoid organs *viz*. spleen, bursa, and thymus-expressed as percentage of live weight of 6 weeks old broiler chicks ranged from 0.084±0.007 (C_1_) to 0.184±0.009 (T_2_), 0.091±0.022 (C_2_) to 0.194±0.058 (T_3_), and 0.329±0.033 (C_2_) to 0.577±0.152 (T_3_), respectively. The relative weight of spleen of broilers in T2 was numerically higher than all the other treatments. The percent weight of bursa and thymus were found to be the highest in the birds of treatment T_3_. No significant (p>0.05) difference was observed on the above parameters between the treated groups. The average weight (percentage of live weight) of liver which has got some role in immunity, ranged from 1.896±0.094 (C_1_) to 2.403±0.152 (T_1_) and showed insignificant (p>0.05) difference between the groups.

**Table-5:** Weight of lymphoid organs (percentage of live weight) of broiler.

Organs	Treatments						p value

	C_1_	C_2_	T_1_	T_2_	T_3_	T_4_	
Spleen	0.084±0.007	0.109±0.011	0.117±0.004	0.184±0.009	0.166±0.061	0.176±0.008	0.09
Bursa	0.188±0.054	0.091±0.022	0.127±0.029	0.161±0.062	0.194±0.058	0.174±0.013	0.56
Thymus	0.517±0.086	0.329±0.033	0.541±0.123	0.365±0.020	0.577±0.152	0.417±0.074	0.38

The weight of lymphoid organs or immune organs of all the treated groups did not differ significantly (p>0.05). Supplement of dietary protein from *Azolla* at 5% or 10% level had no significant effect on the weight of lymphoid organs. Furthermore, enzyme supplementation had no effect on spleen, bursa, and thymus gland relative weights when compared with chick groups fed on the same diet without enzyme supplementation.

### Antioxidant status

The antioxidant enzyme and lipid peroxidation activity in Vencobb broiler birds under different dietary treatments are presented in Table-6. Erythrocyte catalase activity (units/mg of hemoglobin) values of the birds at 6^th^ week of age were 1.27±0.04, 1.34±0.03, 1.90±0.07, 2.05±0.20, 2.94±0.07, and 3.13±0.05 in the treatments C_1_, C_2_, T_1_, T_2_, T_3,_ and T_4_, respectively. Erythrocyte catalase activity was significantly (p<0.05) higher in T_3_ and T_4_ groups compared to all other groups.

**Table-6:** Antioxidant enzyme and lipid peroxidation activity in broiler chicks.

Parameters (unit)	Treatments						p value

	C_1_	C_2_	T_1_	T_2_	T_3_	T_4_	
Erythrocyte catalase (units/mg of hemoglobin)	1.27^c^±0.04	1.34^c^±0.03	1.90^b^±0.07	2.05^b^±0.20	2.94^a^±0.07	3.13^a^±0.05	<0.01
Erythrocyte MDA (nmol MDA/mg of hemoglobin)	2.01±0.15	1.93±0.03	1.95±0.16	1.93±0.12	1.97±0.14	1.92±0.07	0.99

Values bearing different superscripts in a row differ significantly (p<0.05), MDA=Malondialdehyde

The erythrocyte malondialdehyde (MDA) levels (nmol MDA/mg of hemoglobin) of 6-week-old broiler birds under treatments C_1_, C_2_, T_1_, T_2_, T_3,_ and T_4_ were 2.01±0.15, 1.93±0.03, 1.95±0.16, 1.93±0.12, 1.97±0.14, and 1.92±0.07, respectively. The values showed insignificant (p>0.05) difference among the treatment groups with numerically highest and lowest value represented by treatment C_1_ and T_4_, respectively.

## Discussion

The CBH response and antibody titers (log_2_) against SRBC inoculation at 6^th^ week of age of broiler chicks is presented in Table-4. This implied that the CMI response in the *Azolla* supplemented groups was similar as that of the control groups. This is in agreement with the findings of Sujatha *et al*. [17]. The results implied that the SRBC response was better in groups having *Azolla* with enzyme supplementation. This is in agreement with the findings of Prabina and Kumar [18]. Dhumal *et al*. [1] reported that *Azolla* meal feeding in broiler improved the antibody titer values as compared to control group. This further corroborated the findings of Sujatha *et al*. [17] who reported higher mean hemagglutination inhibition titer against GRBC inoculation in the raw *Azolla* fed group than the control group. The weights of lymphoid organs (percentage of live weight) of Vencobb broiler chicks under different dietary treatments are presented in Table-5. The weight of lymphoid organs or immune organs of all the treated groups did not differ significantly (p>0.05). Supplementation of dietary protein from *Azolla* at 5% or 10% level with or without enzyme supplementation had no significant effect on the weight of lymphoid organs. In the available literatures, little evidence has been found regarding the CMI and humoral immune response, as well as, the weight of lymphoid organs in chicken in relation to *Azolla* supplementation in the diet.

The antioxidant enzyme and lipid peroxidation activity in broiler birds under different dietary treatments are presented in Table-6. The erythrocyte catalase level of T_4_ group was found to be significantly higher than the rest of the treated groups except T_3_. Furthermore, erythrocyte catalase levels were found to be significantly (p<0.05) higher than that of control groups. The increase in levels of erythrocyte catalase of broilers fed *Azolla* might be due to presence of iron and copper in *Azolla* as catalase is a heme-containing antioxidant enzyme, which acts sequentially to superoxide dismutase in the conversion of hydrogen peroxide to water [19]. Spears [20] reported that selenium, vitamin E, chromium, cobalt, copper, and vitamin A have immune regulatory properties in cattle.

Fe^+3^ protoporphyrin is the central catalase group. Catalase activity is reduced in Cu deficiency [21]. This is because Cu is necessary to adequate Fe utilization, which is an important component of catalase. Some researchers have reported an increase [22]; others have reported a decrease in erythrocyte catalase activity to be associated with copper deficiency [23]. Erythrocyte MDA (lipid peroxidation activity) levels did not differ significantly (p>0.05) between groups. Vitamins, recognized as a potent lipid-soluble antioxidant, have been found to play a key role in the normal functioning of the immune system and protects against lipid peroxidation of the cell membrane-initiated by free radicals by arresting or entirely preventing the process [24-27]. In spite of high fiber feeding in *Azolla* supplemented groups, due to presence vitamins along with other essential nutrients, no significant level of lipid peroxidation activity was observed in *Azolla* fed groups.

Moreover, the better immune response as observed in the present study might be the due presence of essential nutrients in *Azolla* relating to the immunity of the birds. *Azolla* is rich in protein, contains almost all essential amino acids, carotene, and several growth promoter intermediaries, and minerals such as calcium, phosphorus magnesium, potassium, iron, and copper [1]. Apart from nutrients, *Azolla* also contains certain compounds such as carotenoids, bio-polymers, and probiotics which contribute to higher productivity and health of animals [2].

## Conclusion

From this, it may be concluded that replacement of soybean with *Azolla*, supplying 10% of protein requirement along with enzyme supplementation in the isonitrogenous diet showed better immune response in broiler chickens.

## Authors’ Contributions

This study is a component of the work towards the M. V. Sc. thesis of the first author BC. GPM, CRP, SKM & NCB: Provided guidance during the entire experiment. BC, SKM & KB: Prepared and corrected the manuscript. AD: Helped in conducting the experiment and analysis of immunological parameters. All authors have read and approved the final version of the manuscript.
